# Intra-Individual and Seasonal Variation of Selected Biomarkers for Internal Load Monitoring in U-19 Soccer Players

**DOI:** 10.3389/fphys.2020.00838

**Published:** 2020-08-04

**Authors:** Manuel Becker, Billy Sperlich, Christoph Zinner, Silvia Achtzehn

**Affiliations:** ^1^Institute of Exercise Training and Sport Informatics, German Sport University Cologne, Cologne, Germany; ^2^Decode Dynamics Sports Performance Institute, Waldbronn, Germany; ^3^Integrative and Experimental Exercise Science and Training, Institute of Sport Science, University of Würzburg, Würzburg, Germany; ^4^Department of Sport, University of Applied Sciences for Police and Administration of Hesse, Wiesbaden, Germany; ^5^Institute of Cardiology and Sports Medicine, German Sport University Cologne, Cologne, Germany; ^6^The German Research Centre of Elite Sport, German Sport University Cologne, Cologne, Germany

**Keywords:** biomarker variability, creatine kinase, soccer, youth soccer, internal load, monitoring, point of care testing

## Abstract

The aim of this study was to investigate inter-day and -week as well as intra- and inter-individual variation of selected biomarkers in high-performance youth soccer players to assist practitioners interpreting player’s internal load to counteract underperformance and unwanted health risks. Eleven male youth soccer players were tested multiple times during two 3-week periods at midpoint (3-wk_mid_) and at the end (3-wk_end_) of the first half of a German under-19 1. Bundesliga season. The levels of creatine kinase (CK), urea, and C-reactive protein (CRP) were measured during 3-wk_mid_ and 3-wk_end_ each Monday, Wednesday, and Friday. In 3-wk_mid_ the CK median was 14% higher (241 vs. 212 U/L) compared to 3-wk_end_ (*P* = 0.26, ES = 0.16). Overall, the medians of CK, urea (*P* = 0.59, ES = 0.08), and CRP (*P* = 0.56, ES = 0.10) during 3-wk_mid_ did not differ to the values of 3-wk_end_. Daily coefficient of variations (CVs) ranged from 22 to 71% (CK), 17 to 37% (urea), and 9 to 164% (CRP). Individual medians ranged from 101 to 350 U/L (CK), 23 to 50 mg/dL (urea), and 0.6 to 1.1 mg/L (CRP). High intra-individual variability was demonstrated by large intra-individual CVs (medians: CK 50%, urea 18%, and CRP 45%). Our data show (i) large inter-day and inter-week variability of all biomarkers, depending on the external load and (ii) considerable inter- and intra-individual parameter variations. Creatine kinase concentrations could sensitively reflect soccer-specific loads during the season.

## Introduction

Soccer players under the age of 19 (U-19) are exposed to frequent training sessions and matches, which are scheduled in one- or two-match weeks. It is well known that high match and frequent training session exposure in youth soccer players is associated with increased injury risks (Brink et al., 2010; Pfirrmann et al., 2016).

Monitoring internal and adjusting external load may ensure optimal training and match performances as well as prevent injuries over the course of an entire season. In this regard current expert opinion warrants objective and reliable methods (i) to adjust external load for optimal adaptation and prevention of overuse and (ii) to quantify each player’s fatigue state (Nedelec et al., 2012). From a practical perspective, including that of coaches, monitoring of load designed to detect potential changes in health and performance should be valid, reliable and sensitive, as well as time-efficient, easily applicable, non-fatiguing and as non-invasive as possible (Starling and Lambert, 2018). Finally, dense biomarker monitoring is warranted in order to interpret time-courses of concentrations since most biomarkers are detectable up to several days (Ascensao et al., 2008).

Previous studies with professional soccer players revealed acutely increased creatine kinase (CK), urea, and C-reactive protein (CRP) concentrations induced by soccer-specific loads (de Hoyo et al., 2016; Mohr et al., 2016; Romagnoli et al., 2016) and by various other types of sport (Wiewelhove et al., 2015; Julian et al., 2017). Elevated levels of these markers occur in connection with different types of concentric and eccentric muscle contractions (Banfi et al., 2012). Especially eccentric muscle contractions induce muscle damage and soreness for up to 72 h after the match (Ispirlidis et al., 2008; Fatouros et al., 2010; Nedelec et al., 2012; Thorpe and Sunderland, 2012). Increase in soccer-related muscle damage is often associated with inflammatory response and accompanied by elevation of other blood markers including urea and CRP (Nedelec et al., 2012). With several matches per week and frequent training sessions the concentrations of CK, urea, and CRP might not return to baseline over several weeks.

Therefore, in-season monitoring of CK, urea, and CRP may assist estimating the internal load of muscles, metabolism, and unspecific inflammatory condition (Pepys and Hirschfield, 2003; Brancaccio et al., 2010). Advancements in point-of-care testing (POCT) allow rapid, frequent, and instant evaluation of numerous valid biomarkers for load management, including CK, urea, and CRP (Achtzehn et al., 2018) and dense monitoring may assist coaches in assessing youth player’s load in connection with training and soccer matches (Kunz et al., 2019). Even though dense and frequent monitoring is recommended for load management (Wiewelhove et al., 2015), no study has investigated the intra-individual and seasonal kinetics of selected biomarkers in high-performance U-19 soccer players every second or third day for several weeks.

This exploratory study aimed to compare two selected time periods within a national U-19 soccer season to investigate (i) the inter- and intra-individual kinetics of selected biomarkers and (ii) the seasonal inter-day and inter-week variability of CK, urea, and CRP. From a practical perspective the study results should assist assessing high-performance youth soccer players’ internal load to counteract underperformance and unwanted health risks.

## Materials and Methods

### Participants

Eleven male youth soccer players of a German U-19 first league (1. Bundesliga) team were tested (18 ± 1 yrs; 180 ± 10 cm; 72 ± 6 kg) during two 3-wk periods. Table 1 summarizes important performance variables of each player at the beginning of the season.

**TABLE 1:** Selected performance variables of each players at the beginning of the season.

Player ID	Position	YoYo intermittent recovery test Level 2 (m)	Running speed at 4 mmol/L Blood lactate during incremental testing (m/s)	Time 30m-Sprint (s)
1	d mid	1,120	3.85	4.34
2	d mid	1,220	4.01	n.d.
3	for	1,020	3.85	4.11
4	def	1,380	3.77	4.28
5	o mid	800	3.67	3.97
6	for	1,220	3.92	3.99
7	def	1,180	3.73	4.12
8	d mid	1,300	3.9	4.34
9	mid	1,260	3.84	4.14
10	mid	1,260	3.46	4.06
11	o mid	n.d.	4.21	4.00
Team mean		1,176 ± 164	3.83 ± 0.19	4.13 (0.14)

*def, defender; d mid, defensive midfielder; mid, midfielder; o mid, offensive midfielder; for, forward; n.d., not determined.*

Two players were members of the U-19 national teams of Germany and Poland. We investigated only field players since physical requirements vary widely between field players and goalkeepers. All players were informed about all testing and signed an informed consent. This study was approved by the ethics committee of the German Sport University (Cologne, Germany) and was conducted according to the Declaration of Helsinki.

### Research Design

**FIGURE 1:**
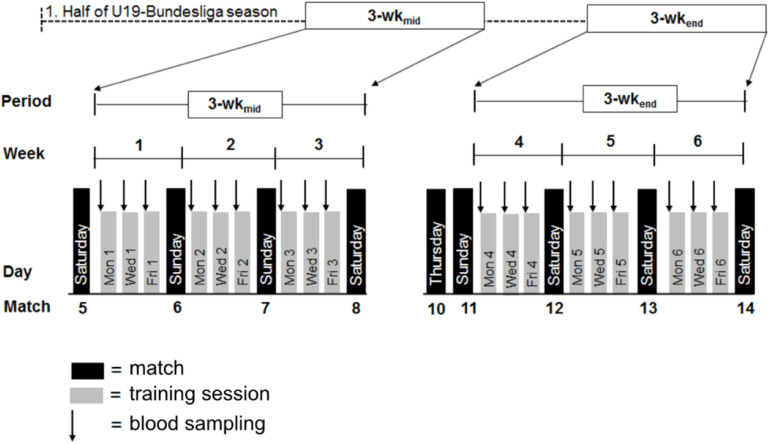
Schematic illustration of the study design with all procedures. Black arrows indicate weekdays of blood sampling (on every Monday, Wednesday, and Friday).

All data were obtained on 18 testing days in two 3-wks periods, i.e., at midpoint (3-wk_mid_) and at the end of the first half of the season (3-wk_end_) during a German U-19 first league season. A total of 3-wk_mid_ took place between match 5 and 8, 3-wk_end_ between match 10 and 14, which was at the end of the first half season (Figure 1). Due to the teams’ scheduling of training sessions and camps as well as administrative difficulties it was not possible to start the investigation at the beginning of the season as initially intended.

Before and after each week of 3-wk_mid_ and 3-wk_end_, the participants played one U-19 first league match on either Saturday or Sunday (kickoff between 11.00 a.m. and 1.00 p.m.). Two first league matches were scheduled before the 3-wk_end_ (rounds 10 and 11 were played on Thursday 6.30 p.m. and Sunday 1.00 p.m.).

Tables 2A,B summarize the main content of every training session including session duration and intensity, external load and the responses of CK, urea, and CRP. The intensity zones (1 = easy, 2 = moderate, and 3 = hard) were set according to the coach’s subjective rating of intensity. External load was defined as the time spent in each intensity zone multiplied with the coach’s subjective ranking (i.e., time in zone 1 × 1, time in zone 2 × 2, and time in zone 3 × 3). The players with more than 45-min match participation were defined as starters, players with less than 45-min match participation as substitutes.

**TABLE 2:** Day of testing, session duration, main content, intensity, external load, and the responses of creatine kinase, urea, and C-reactive protein (CRP).

(A)	Analysis of 3-wk_mid_												
Day	Session duration (min)	Main content	Intensity 1/2/3 (min)	External load (a.u.)	Creatine kinase (U/L)			Urea (mg/dL)			CRP (mg/L)		
					Median (n)/starter (*n*)/substitutes (*n*)	Min/Max	CV (%)	Median (*n*)/starter (*n*)/substitutes (*n*)	Min/Max	CV (%)	Median (*n*)/starter (*n*)/substitutes (*n*)	Min/Max	CV (%)
Sat_pre_	54 + 35	Match + warm up and cool-down	10/25/54	222									
Sun_pre_	0	No training	0/0/0	0									
Mon1	85	Strength, recovery	40/30/15	145	229 (11) 364 (7)/162 (4)	76/645	59	30 (11) 30 (7)/30 (4)	20/63	37	0.7 (10) 1.0 (6)/0.7 (4)	0.5/3.1	76
Tue1	80	Tactical drills, training match	30/30/20	150									
Wed1	95	strength, speed, tactical drills	30/15/50	210	264 (8) 252 (6)/273 (2)	168/465	32	47 (8) 46 (6)/47 (2)	26/55	24	0.7 (7) 0.7 (5)/0.8 (2)	0.5/1.3	32
Thu1	0	No training	0/0/0	0									
Fri1	60	Tactical drills, training match	15/20/25	130	166 (9) 174 (6)/164 (3)	74/323	41	33 (9) 30 (6)/40 (3)	22/58	34	0.7 (6) 0.7 (4)/0.7 (2)	0.5/1.0	23
Sat1	55	Tactical drills, speed, training match	15/25/15	110									
Sun1	45 + 35	Match + warm up and cool-down	10/25/45	195									
Mon2	85	Tactical drills, recovery	50/30/5	125	337 (10) 381 (6)/233 (4)	77/511	44	36 (10) 36 (6)/38 (4)	20/65	36	1.2 (8) 1.5 (6)/0.5 (2)	0.5/4.5	83
Tue2	30	Strength	15/5/10	55									
	75	Tactical drills	30/30/15	135									
Wed2	45	Strength, speed	5/5/35	120	289 (9) 276 (5)/374 (4)	149/544	36	34 (9) 27 (5)/40 (4)	20/50	34	0.9 (6) 0.6 (3)/1.1 (3)	0.6/2.2	54
	55	Tactical drills, recovery	30/10/15	95									
Thu2	0	No training	0/0/0	0									
Fri2	60	Tactical drills, training match	15/20/25	130	171 (8) 171 (4)/196 (4)	101/366	39	34 (8) 25 (4)/40 (4)	20/46	30	0.6 (8) 0.6 (4)/0.6 (4)	0.5/1.0	23
Sat2	55	Tactical drills, speed, training match	15/25/15	110									
Sun2	44 + 35	Match + warm up and cool-down	10/25/44	192									
Mon3	55	Recovery	40/15/0	70	299 (9) 310 (5)/253 (4)	147/665	44	42 (9) 43 (5)/39 (4)	22/71	34	0.8 (8) 0.9 (4)/0.7 (4)	0.5/1.8	45
Tue3	30	Strength	15/5/10	55									
	75	Tactical drills	30/30/15	135									
Wed3	50	Strength, speed	5/0/45	140	368 (6) 329 (3)/406 (3)	227/575	31	32 (6) 42 (3)/27 (3)	27/50	25	0.7 (5) 0.7 (3)/1.9 (2)	0.6/2.3	57
Thu3	60	Tactical drills, training match	15/20/25	130									
Fri3	55	Tactical drills, speed, training match	15/25/15	110	219 (9) 219 (5)/270 (4)	177/569	42	33 (9) 38 (5)/32 (4)	20/50	28	0.9 (7) 0.6 (3)/1.2 (4)	0.6/1.3	31
Sat3	52 + 35	Match + warm up and cool-down	10/25/52	216									
Sun3	0	No training	0/0/0	0									
Thu_pre_	54 + 35	Match + warm up and cool-down	10/25/54	222									
Fri_pre_	55	Tactical drills, recovery	45/10/0	65									
Sat_pre_	55	Tactical drills, speed, training match	15/25/15	110									
Sun_pre_	39 + 35	Match + warm up and cool-down	10/25/39	177									
Mon4	50	Tactical drills, recovery	40/10/0	60	204 (10) 576 (4)/168 (6)	97/791	71	41 (10) 37 (4)/43 (6)	27/59	20	1 (10) 1.1 (4)/0.9 (6)	0.6/1.8	40
Tue4	0	No training	0/0/0	0									
Wed4	65	Strength, speed, tactical drills	15/15/35	150	135 (8) 180 (3)/115 (5)	55/213	40	31 (8) 23 (3)/31 (5)	20/43	24	0.9 (3) 0.9 (2)/1.0 (1)	0.8/1.0	9
	70	Tactical drills, training match	30/15/25	135									
Thu4	70	Tactical drills, training match	70/20/20	170									
Fri4	55	Tactical drills, speed, training match	15/25/15	110	323 (10) 376 (4)/236 (6)	212/555	29	40 (10) 30 (4)/46 (6)	21/50	26	– (0)	–	–
Sat4	47 + 35	Match + warm up and cool-down	10/25/47	201									
Sun4	25	Recovery	25/0/0	25									
Mon5	70	Strength, tactical drills, recovery	45/15/10	105	152 (9) 193 (4)/133 (5)	54/307	46	38 (9) 38 (4)/40 (5)	23/57	26	0.6 (9) 0.5 (4)/1.1 (5)	0.5/3.2	79
Tue5	90	Tactical drills, training match	30/25/35	185									
Wed5	60	Strength, speed	15/5/40	145	274 (7) 293 (3)/233 (4)	175/464	34	36 (7) 42 (3)/33 (4)	26/50	21	0.7 (7) 0.7 (3)/1.1 (4)	0.5/11.0	157
Thu5	55	Tactical drills, training match	15/5/35	130									
Fri5	55	Tactical drills, speed, training match	15/25/15	110	306 (7) 281 (3)/317 (4)	148/352	22	43 (7) 44 (3)/37 (4)	30/54	22	0.5 (7) 0.5 (3)/0.8 (4)	0.5/9.1	164
Sat5	44 + 35	Match + warm up and cool-down	10/25/44	192									
Sun5	0	No training	0/0/0	0									
Mon6	60	Strength, tactical drills, recovery	45/15/0	75	165 (8) 231 (5)/117 (3)	66/296	43	33 (8) 34 (5)/29 (3)	23/60	33	0.5 (8) 0.5 (5)/0.5 (3)	0.5/3.3	94
Tue6	65	Strength, speed, tactical drills	15/15/35	150									
	75	Tactical drills, training match	25/20/30	155									
Wed6	0	No training	0/0/0	0	– (0)	–/–	–	– (0)	–/–	–	– (0)	–/–	–
Thu6	0	No training	0/0/0	0									
Fri6	45	Tactical drills, speed, training match	15/15/15	90	111 (8) 104 (5)/118 (3)	56/230	40	35 (8) 35 (5)/32 (3)	21/40	17	0.6 (8) 0.5 (5)/0.6 (3)	0.5/2.1	66
Sat6	48 + 35	Match + warm up and cool-down	10/25/48	204									
Sun6	0	Beginning of winter break	0/0/0	0									

*Blood sampling took place before each training session, reflecting the training load on the days before. The intensity zones (1 = easy, 2 = moderate, and 3 = hard) were set according to the coach’s subjective rating. Players with more than 45-min match participation were defined as starters, players with less than 45 min match participation as substitutes.*

Because of the observational nature of this study, data were analyzed retrospectively. Therefore, the present results had no immediate consequences for coaches and medical staff to adjust training load, medication or recovery strategies by coaches or the medical staff.

### Blood Sampling and Processing

During 3-wk_mid_ and 3-wk_end_ on every Monday (Mon), Wednesday (Wed), and Friday (Fri) capillary blood was sampled from the participants’ earlobe before the beginning of each training session and analyzed immediately after sampling. Training sessions commenced at 5.00 p.m on Mon, at 11.30 a.m. on Wed, and at 3.00 p.m. on Fri. Only on Wed1 (i.e., the first Wednesday for data collection) the training began at 5.30 p.m. On Wed6 (i.e., the 6th Wednesday for data collection) all players had one day off, so all testing was canceled by the coaches.

Creatine kinase and urea concentrations in each 32 μL blood samples were analyzed employing Reflotron^®^ Plus system (Roche Diagnostics, Mannheim, Germany). The measuring ranges were 24.4 to 1,700 U/L (CK) and 20.0 to 300.0 mg/dL (urea). The intra-assay coefficient of variation (CV) (test-retest reliability) for CK was 3.5% and for urea ≤3.0%. The inter-assay CV (day-to-day reliability) for CK was ≤4.2% and for urea ≤3.5%. QuikRead go^®^ hsCRP + Hb tests for a QuikRead go^®^ photometer (Orion Diagnostica, Espoo, Finland) determined CRP concentration from 20 μL capillary blood sample. During the last two weeks of the study QuikRead go^®^ wrCRP + Hb tests were used, whereby only 10 μL of blood were needed. Measuring ranges were 0.50 to 75.00 mg/L (hsCRP), and 0.50 to 300.00 mg/L (wrCRP), respectively. The intra-assay CV for hsCRP ≤ 5.9% and for wrCRP ≤ 4.9%. The inter-assay CV for hsCRP ≤ 2.7% and for wrCRP ≤ 4.8%.

### Statistical Analyses

Data distribution with Kolmogorov–Smirnov and the Shapiro–Wilk test showed no normal distribution of CK (*P* = 0.001 and *P* < 0.001, respectively), CRP (*P* < 0.001 and *P* < 0.001, respectively), and urea (*P* < 0.001 and *P* < 0.001, respectively) concentrations. Therefore, all data are presented in box plots as medians, 25 to 75% percentiles as well as minimum and maximum values. Inter-day and inter-week variability are demonstrated by boxplots which represent the results from every testing day. Intra- and inter-individual variability are demonstrated by boxplots representing the results from every player during the observation period. The CV quantified the parameter variability and was defined as standard deviation divided by mean value multiplied by 100. CV was determined for all biomarkers during the complete study process as well as for 3-wk_mid_ and 3-wk_end_ and for all testing days. CV was calculated from the individual parameter responses in the study process to quantify intra-individual variability. The differences between 3-wk_mid_ and 3-wk_end_ were assessed employing Wilcoxon comparison. The effect size (ES) measurement for non-parametric tests (*r* = Z/√N) (Rosenthal, 1994) were calculated between 3-wk_mid_ and 3-wk_end_. The level of significance was *P* ≤ 0.05. The analyses were carried out with SPSS Statistics 24 (IBM, Chicago, IL, United States) and Excel 2010 (Microsoft Corp., Redmond, WA, United States), respectively.

## Results

### Biomarker Responses During 3-wk_mid_ and 3-wk_end_

**FIGURE 2:**
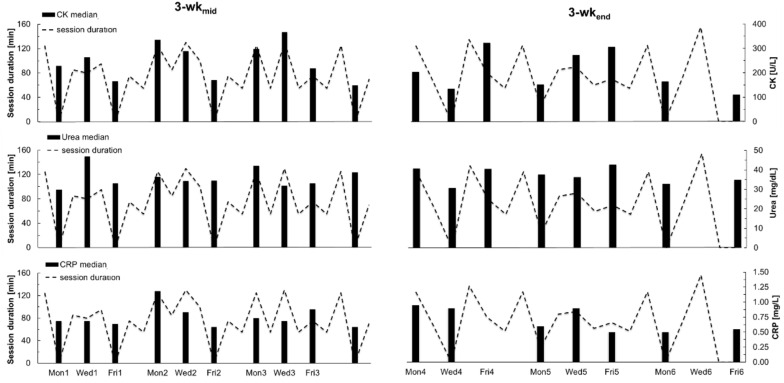
Median variation of creatine kinase, urea and C-reactive protein and session duration (dotted lines) during both observation periods (3-wk_mid_ and 3-wk_end_).

Figure 2 illustrates the median variation of creatine kinase, urea and CRP and session duration during both observation periods (3-wk_mid_ and 3-wk_end_).

Table 3 shows the median as well as minimum and maximum values and CVs of CK, urea, and CRP during 3-wk_mid_ and 3-wk_end_. Overall, the medians of CK (*P* = 0.26, ES = 0.16), urea (*P* = 0.59, ES = 0.08), and CRP (*P* = 0.56, ES = 0.10) in 3-wk_mid_ did not differ compared to the values of 3-wk_end_.

**TABLE 3:** Number of samples, median, minimum, and maximum as well as coefficients of variation of creatine kinase, urea, and C-reactive protein in both observation periods (3-wk_mid_ and 3-wk_end_).

	Creatine kinase (U/L)		Urea (mg/dL)		CRP (mg/L)	
	*3-wk_mid_*	3-wk_end_	*3-wk_mid_*	3-wk_end_	*3-wk_mid_*	3-wk_end_
Number of samples (*n*)	87	59	87	59	72	44
Median	241	212	36	36	0.7	0.8
Minimum/maximum	47/665	54/791	20/71	20/60	0.5/4.5	0.5/11
Coefficients of variation (%)	50	58	33	26	68	147

The external load did not differ between 3-wk_mid_ and 3-wk_end_ (*P* = 0.80; ES = 0.06).

During both observation periods the biomarkers ranged from 47 to 791 U/L (CK), 20 to 71 mg/dL (urea), and 0.5 to 11.0 mg/L (CRP), respectively. CVs ranged from 33 (urea, 3-wk_mid_) to 147% (CRP, 3-wk_end_).

Results from Fri6 were excluded in Tables 2A,B because all players had two successive days off on the previous days and consequently external load was different before Fri6 compared to the other testing days. Therefore, 146 CK and urea as well as 116 CRP tests were analyzed in Tables 2A,B.

### Seasonal Inter-Day and Inter-Week Variability and Correlation of CK, Urea, and CRP

**FIGURE 3:**
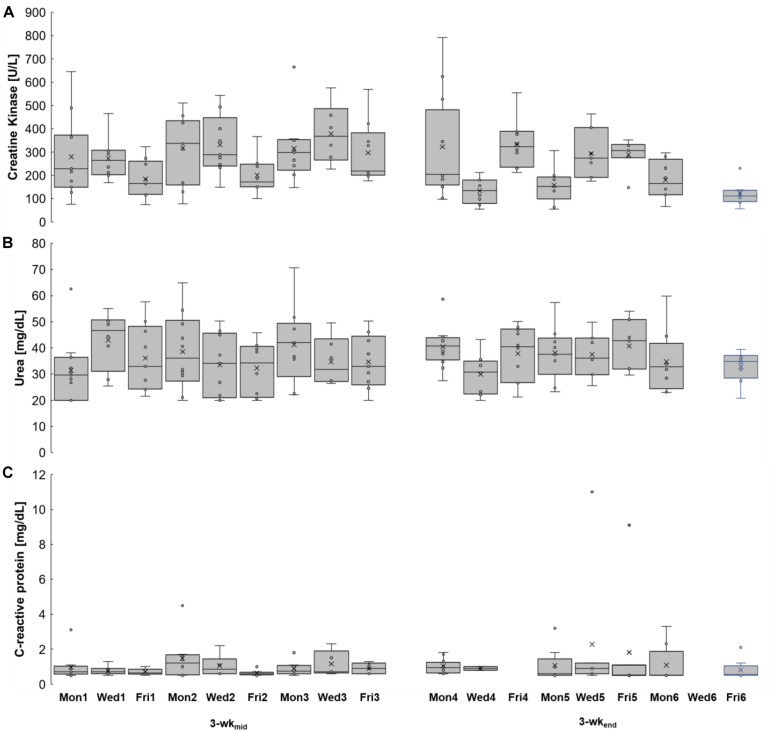
Kinetics of creatine kinase **(A)**, urea **(B)**, and C-reactive protein **(C)** levels during 3-wk_mid_ and 3-wk_end_. Left column: 3-wk_mid_. Right column: 3-wk_end_. Box = 25 to 75% percentile; line inside the box = median; x = mean; whisker = lowest and greatest value, excluding outliers and extreme values; circle = outlier; asterisk = extreme value. Mon1–6, Wed1–6, Fri1–6 = Monday, Wednesday, Friday in study week 1–6.

Figure 3 and Tables 2A,B summarize the inter-day (e.g., Mon1 vs. Wed1 vs. Fri1) and inter-week (e.g., Mon1 vs. Mon2 vs. Mon3) variability of CK, urea, and CRP. In 3-wk_mid_ CK medians were higher every Mon and Wed compared to the values on Fri (e.g., Mon1: 229 U/L (+ 38%), Wed1: 264 U/L (+ 59%), and Fri1: 166 U/L). CRP medians in week 1 and 2 were also higher on Mon and Wed compared to Fri (e.g., Mon2: 1.2 mg/L (+ 100%), Wed2: 0.9 mg/L (+ 42%), and Fri2: 0.6 mg/L). In 3-wk_mid_ daily mean CK and CRP values showed very large correlation (*r* = 0.87; *P* = 0.001, *n* = 10). In 3-wk_end_ daily mean CK and CRP values showed large correlation but not significant (*r* = 0.64; *P* = 0.119, *n* = 7).

In 3-wk_end_ urea medians on Fri4 (41 mg/dL, + 31%) and Fri5 (43 mg/dL, + 18%) were higher than on Wed4 (31 mg/dL) and Wed5 (36 mg/dL), respectively. Daily CVs ranged from 22% (Fri5) to 71% (Mon4) for CK, from 17% (Fri6) to 37% (Mon1) for urea and from 9 (Wed4) to 164% (Fri5) for CRP concentrations.

One day after the matches (Mon2 and Mon3) median CK values (337 and 299 U/L) were about 70% higher than 2 days postmatch (Mon1, Mon4, Mon5, and Mon6) with CK medians ranging from 152 to 229 U/L.

One day after match day median CRP values (1.2 and 0.8 mg/L) were about 43% higher than after 2 days (range from 0.5 to 1.0 mg/L), while median urea values were nearly unchanged between one (36 and 34 mg/dL) and 2 days (range from 30 to 41 mg/dL) postmatch.

### Inter- and Intra-Individual Variability of CK, Urea, and CRP

**FIGURE 4:**
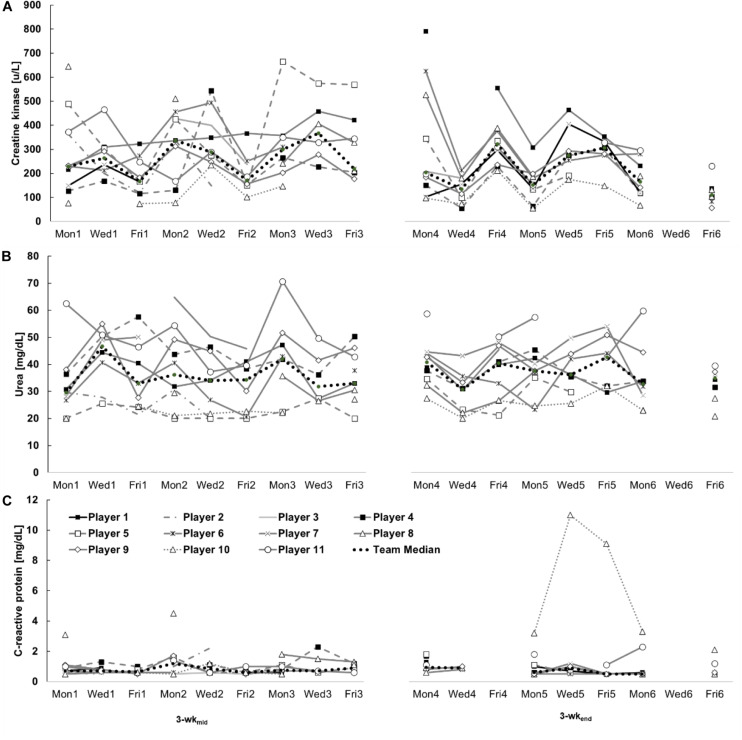
Intra-individual creatine kinase **(A)**, urea **(B)**, and C-reactive protein **(C)** kinetics of eleven youth soccer players and the team’s median during two investigation periods (3-wk_mid_ and 3-wk_end_). Mon1 - Fri3 = 3-wk_mid_, Mon4 - Fri6 = 3-wk_end_.

**FIGURE 5:**
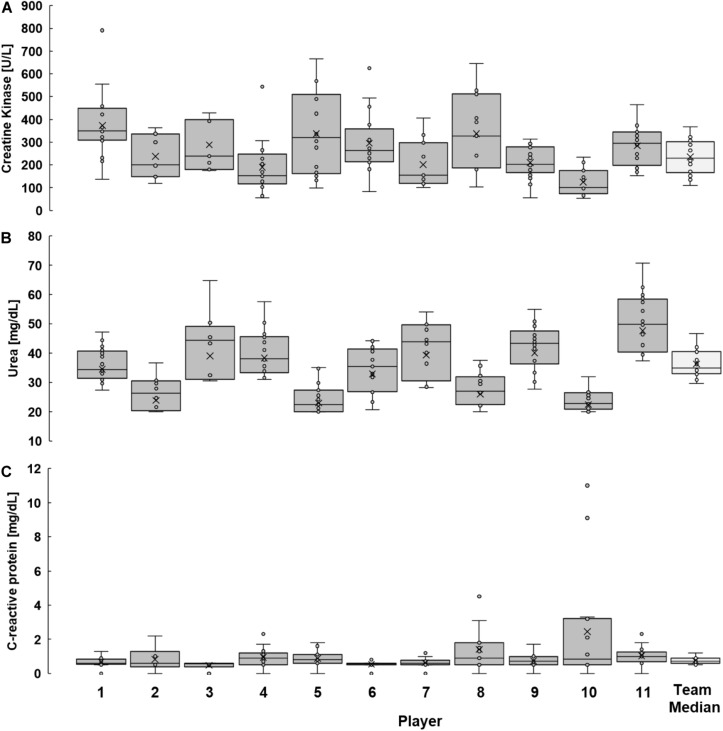
Individual distribution of creatine kinase **(A)**, urea **(B)**, and C-reactive protein **(C)** responses to training and match loads during this study. Illustrated are the results of all players during the observation period as well as the median values of the team. (Box = 25–75% percentile; x = mean; line inside the box = median; whisker = lowest and greatest value, excluding outliers and extreme values; circle = outlier and extreme values.

The individual kinetics in Figure 4 and the box plots in Figure 5 illustrate the inter- and intra-individual variability of all biomarkers. Individual median values for CK responses ranged from 101 (player 10) to 350 U/L (player 1), for urea responses from 23 (player 5) to 50 mg/dL (player 11) and for CRP responses from 0.6 (player 1, 6, and 7) to 1.1 mg/L (player 8 and 10).

Individual CVs in Table 4 show a high inter-individual variation (CK: 33–64%, urea: 14–22%, and CRP: 15–136%). High intra-individual variability is underlined by the broad range of each player’s values [e.g., player 5 (CK): 98–665 U/L, player 9 (urea): 28–55 mg/dL, player 10 (CRP): 0.5–11.0 mg/L], validated by the team’s median CV listed in Table 3 (CK: 50%, urea: 18%, and CRP: 45%).

**TABLE 4:** Creatine kinase, urea, and C-reactive protein responses of each player.

	Creatine kinase (U/L)				Urea (mg/dL)				C-reactive protein (mg/L)			
Player ID	Number of samples	Median	Min/Max	CV (%)	(*n*)	Median	Min/Max	CV (%)	(*n*)	Median	Min/Max	CV (%)
1 (d mid)	16	350	137/791	40	16	35	27/47	15	11	0.6	0.5/1.3	36
2 (d mid)	7	200	119/364	36	7	28	20/37	23	5	0.6	0.2/2.2	80
3 (for)	7	238	175/428	38	7	45	31/65	25	5	0.6	0.5/0.6	10
4 (def)	17	153	55/544	63	17	38	31/58	19	14	0.9	0.5/2.3	53
5 (o mid)	14	320	98/665	55	14	23	20/35	22	10	0.9	0.6/1.8	45
6 (for)	16	264	83/625	46	16	36	21/44	21	13	0.6	0.5/0.8	15
7 (def)	11	155	102/405	52	11	45	28/54	18	9	0.6	0.5/1.2	37
8 (d mid)	11	328	104/645	44	11	27	20/38	18	10	1.1	0.5/4.5	81
9 (mid)	17	203	56/313	34	17	44	28/55	18	14	0.7	0.5/1.7	43
10 (mid)	15	101	54/234	48	15	23	20/32	14	13	1.1	0.5/11.0	134
11 (o mid)	15	296	152/465	30	15	50	37/71	19	13	1.0	0.6/2.3	46
Team median	15	238	102/465	44	15	36	27/47	19	11	0.7	0.5/1.8	45

*def, defender; d mid, defensive midfielder; mid, midfielder; o mid, offensive midfielder; for, forward.*

To illustrate inter- and intra-individual variability, only players with at least 11 analyzed blood tests were included.

## Discussion

The aim of the present study was to compare the results of two selected time periods within a soccer season, to investigate the inter-day and inter-week variability of selected biomarkers (CK, urea, and CRP) and to investigate the inter- and intra-individual kinetics of each variable. The main findings of this study were: (i) large inter-day and inter-week variability of all biomarkers and (ii) considerable inter- and intra-individual parameter variations.

### Inter-Day Variability of CK, Urea, and CRP

Here we measured large inter-day variability of CK, urea, and CRP concentrations in youth players during two competition 3-wk periods. Based on the large variability we conclude that testing of CK, urea, and CRP should be performed as frequently as possible to (i) understand the kinetics of each variable during a season and (ii) generate meaningful consequences for training and match tactics. For example, one blood test per week may assess the current status of CK, urea, and CRP, but single sampling prevents to judge whether the concentrations are increasing, peaking or decreasing. Therefore, when applying single measurements, e.g., one test each week, the potential for data interpretation – and consequently for practical consequences – is severely limited (as shown by the inter-day variation in Figure 5). Therefore, biomarker analyses should be implemented as a monitoring tool for trend diagnosis to assess the kinetics, and not only as status diagnosis. Sperlich et al. (2016) conducted day-to-day CK and urea tests during a 3-wk altitude training camp of national distance runners, while others (Hartmann and Mester, 2000) conducted dense urea monitoring in a single case study (type of sport is unknown) for approximately 4 weeks. Both studies also revealed large inter-day and intra- and inter-individual variability.

By viewing the CK-kinetics during the testing weeks one, two and three, it seems the coach’s load programming was mirrored by CK since (as shown in Tables 2A,B) in each of these weeks the most intense training loads took place during the Tuesday and Wednesday sessions, with reduced training loads on Thursdays and Fridays. Consequently on every Monday (post-match) and Wednesday CK concentrations reached higher levels than the respective Friday value, indicating lower muscle stress or greater in-week adaptation at the end of the week.

When interpreting results, the potential overlap of acute and chronic changes should be considered (Meister et al., 2014). The CK inter-day variability of the present study may be attributed to different external loads between each day and to the number of training sessions per day (Ehlers et al., 2002). CK accumulations after repeated load stimuli (Mohr et al., 2016) and muscular adaptions in the study progress (Brancaccio et al., 2010) can be further reasons for inter-day variability. The highest CK median of the present study (Wed3: 368 U/L) may also demonstrate the relationship between various load stimuli and CK variations: one day before the blood sampling on Wed3, players completed two training sessions (total training duration: 105 min) with 25 min of intense loading (Tables 2A,B). Additionally, three days before blood sampling a U-19 first league match took place, which may have also contributed to the CK accumulations (Mohr et al., 2016).

Alternation of match days between Saturday (test time: two days postmatch) and Sunday (one day postmatch) impacts CK responses on Mondays at the beginning of the training week, especially for the players with more than 45 min match participation. As seen in Tables 2A,B CK results after Sunday matches (on Mon2, Mon3, and Mon4) have shown higher values for the starting players compared to Saturday match days.

Overall, it seems that youth soccer players need approximately 24–48 h to recover after an official (Djaoui et al., 2016) or simulated match (Kunz et al., 2019). Thus, we recommend structuring the external load accordingly.

Urea inter-day variability is affected by load intensity and duration (Haralambie and Berg, 1976). Further influencing factors are fluid and protein intake (Hartmann and Mester, 2000), as well as the adaptation of protein metabolism to exercise. Moreover, urea accumulations after repeated load stimuli should be considered. In the present study, the highest urea median in 3-wk_end_ (Fri5: 43 mg/dL) was reached after three successive training days. As seen in Table 2, on each of these 3 days the players were exposed to exceptionally high training loads, and as mentioned earlier (Haralambie and Berg, 1976), the repetition of high training loads 3 days in a row most probably explains the elevated protein catabolism.

The CRP inter-day variability shown in this study can be explained by the differing load stimuli (Tables 2A,B) in the daily training routine (Chatzinikolaou et al., 2010). Further, elevated CRP concentrations could be a result of tissue damage derived from body contacts (Singh et al., 2011), partly explaining the observed CRP variability. Injury and illness (Vermeire et al., 2006), as well as person-specific characteristics like blood pressure and total cholesterol could potentially affect CRP values (Donges et al., 2010). impact CK responses on Mondays at the beginning of the training week. Especially CK results after Sunday matches (on Mon2 and Mon3) have shown higher values compared to Saturday match days.

### Inter-Week Variability of CK, Urea, and CRP

Considerable differences between CK, urea, and CRP responses were found in successive testing weeks (e.g., Wed1 vs. Wed2 vs. Wed3). To the best of our knowledge no other publication U-19 soccer players has examined the inter-week variability of selected biomarkers by frequent blood sampling. In the present study, periodical CK variations were shown in the first three testing weeks, which are most probably a result of the varying external loads as seen in Tables 2A,B. The CK-kinetics in 3-wk_mid_ reflected a certain periodization model planned by the coach’s external load through increasing loads in three successive weeks. This observation is supported by elevated median values 3 weeks in a row based on the weekly average, and by the Wednesday results, showing that soccer-specific loads are sensitively reflected by the players’ CK levels when frequent and dense measures are performed. In contrast, no periodical CK variations were shown in the last three testing weeks. This could be argued with the fact, that the weekly training plans in 3-wk_end_ showed an altered structure compared to 3-wk_mid_. For instance, in 3-wk_end_ the days with no training sessions varied from week to week (Tue4, Sun5, Wed6, and Thu6).

Based on the present data we assume that CK concentrations reflect changes in the training load during a U-19 first league season. The relationship between the intended load management and the players’ CK responses are confirmed by previous data (Malone et al., 2018) showing a relationship between systematically monitored CK concentrations and the expectable individual performance output in the following training sessions. Furthermore, we identified high correlations in 3-wk_mid_ and 3-wk_end_ between the mean daily CK and CRP values (*r* = 0.87; *P* = 0.001 and *r* = 0,64; *P* = 0.12), which can be explained by the fact that muscle damage is often accompanied by local inflammatory responses (Singh et al., 2011).

Higher urea medians on Fri4 and Fri5 compared to Wed4 and Wed5 were noticeable, indicating accumulated metabolic stress at the end of the week. One reason can be that in 3-wk_end_ training loads were most intense on Wednesdays and Thursdays, which was consequently reflected in higher Friday values.

### Inter- and Intra-Individual Variability of CK, Urea, and CRP

The present data demonstrates a large inter-individual variability of CK, urea, and CRP concentrations. Thus, we could verify the necessity of intra-individual reference values for interpreting parameter results. Universal soccer-specific reference values (Lazarim et al., 2009) are indeed useful for general classifications of results, but the validity is low for a player’s individual stress assessment and the subsequent load management. Individualized interpretations have already been recommended for many years (Hartmann and Mester, 2000).

The results of this study revealed a considerable intra-individual variability, clearly visible in the CVs of CK and CRP. The latter study identified CVs of 40% (CK), 13% (urea), and 29% (CRP), respectively. Sperlich et al. (2016) and Hartmann and Mester (2000) also found high inter- and intra-individual CK and urea variations in runners during a 3-wk training camp, and in one athlete where the type of sport was unknown during a 4-wk training camp, respectively. The varying external loads shown in Tables 2A,B maybe one factor for the large inter- and intra-individual variability, because the concentrations were higher after days with high external loads compared to days with lower external loads. For instance, CK concentration on Mon2 (195 a.u., 337 U/L) was higher compared to Mon5 (25 a.u., 152 U/L). In addition, the already discussed factors of inter-day variability, as well as person-specific characteristics may affect inter- and intra-individual CK variations, including individual physical performance levels (Ehlers et al., 2002), various player positions and the variably pronounced CK activity in high and low responders (Brancaccio et al., 2010) and of course the training load. Genetic predisposition is another factor (Meltzer, 1971) which may explain the variability. Inter- and intra-individual urea variability may be influenced by external factors (as discussed for inter-day variability) and by person-specific characteristics, including various player positions and the players’ individual response behavior to physical strains.

### Practical Relevance

Because of the practical advantages of this method (simple application, small sample volume, and rapid result reporting), POCT seems to be a practical method to monitor CK, urea, and CRP to judge high-performance U-19 players’ individual physical stress responses. Internal biomarker analyses provide evidence for the recovery status of biological systems, which cannot be analyzed by other external monitoring procedures (e.g., heart rate measurement, GPS tracking, and subjective mood assessment). Consequently, in accordance with others (Wiewelhove et al., 2015), we recommend to combine markers representing various mechanisms contributing to fatigue, e.g., neuro-muscular performance measurements (i.e., jump performance) and perceived muscle fatigue (e.g., delayed onset of muscle soreness, DOMS).

Because CK, urea, and CRP showed a large inter-day variability we advise the assessment of all variables as frequently and densely as possible in order to identify trends in their kinetics. For this reason, our recommendation is not to assess the biomarkers within a (single) status diagnostic procedure but rather by frequent in-season monitoring. CK, urea, and CRP concentrations should be interpreted by intra-individual kinetics and not by general sport-specific cut-off references. The CK concentrations seem to respond sensitively to soccer-specific loads. CK concentrations offer interesting and additional information which can be utilized to optimize the soccer player’s individual internal load, which may allow the prevention of fatigue-induced underperformances and injury.

Because CRP detectability in blood is shorter compared to that of CK (which is a practical disadvantage), and because CRP seems to be less sensitive than CK in reflecting external loading, our recommendation is not to frequently assess CRP levels. The possibility to assess CRP promptly and effortlessly with capillary blood is becoming increasingly important in connection with health screening in the event of complaints from athletes. Urea concentrations appear insufficient to claim valid inferences about the players’ acute stress levels, therefore urea isn’t an appropriate marker for the acute assessment of internal load but it could be an appropriate marker for assessing long-term (chronical) metabolic stress.

### Limitations

Due to the players’ training schedule more standardized time points for sampling were not feasible. In addition to the blood sampling the session ratings of perceived exertion (sRPE) method seems a valid method of quantitating exercise training during a wide variety of types of exercise (Foster et al., 2001). In the present investigation, the participants were among the top-ranked youth players in Germany, encountering a great amount of competitive stress. Participants are wanting to qualify for national team ranking or professional player status. In our experience and also mentioned elsewhere (Borg, 1998) highly motivated participants may underestimate perceived exertion in comparison with their individual work capacity. Since the aim of this study was to obtain objective variables, we refrained from employing the sRPE method.

## Conclusion

Because of the high intra-individual variability of CK, urea, and CRP concentrations, testing of CK, urea and CRP should be performed as frequently as possible in order to (i) understand the kinetics of each variable during a season in relation to the given training load, and (ii) generate meaningful consequences for training and match tactics. Our data showed lower CK values toward the end of the half season. Therefore, we conclude that CK should be assessed with individual reference values compared to the midpoint of the U-19 first league half season. CK sensitively reflected soccer-specific loads during our observation suggesting this variable as a promising biomarker for internal load estimation when assessed frequently.

## Data Availability Statement

The datasets generated for this study are available on request to the corresponding author.

## Ethics Statement

This study was approved by the ethics committee of the German Sport University (Cologne, Germany). Written informed consent to participate in this study was provided by the participants’ legal guardian/next of kin.

## Author Contributions

All authors listed have made a substantial, direct and intellectual contribution to the work, and approved it for publication.

## Conflict of Interest

The authors declare that the research was conducted in the absence of any commercial or financial relationships that could be construed as a potential conflict of interest.
